# Supplementary material for: Mental health during the COVID-19 pandemic: Impacts of disease, social isolation, and financial stressors
Source: PLoS One. 2022 Nov 23;17(11):e0277562. doi: 10.1371/journal.pone.0277562 (PMC9683625; doi:10.1371/journal.pone.0277562)
Supplement: S1 Table — (PDF) [file pone.0277562.s001.pdf]

	N	Unweighted proportion (%)	Weighted proportion (%)	Census proportion (%)
All responses	11,974,779			
Gender				
Female	7,863,065	67.1%	53%	50.8%
Male	3,863,208	32.9%	47%	49.2%
Age				
18-24	743,700	6.2%	10.9%	11.9%
25-34	2,130,505	17.8%	16.7%	17.9%
35-44	2,324,455	19.5%	16.6%	16.4%
45-54	2,202,913	18.5%	17.9%	16.0%
55-64	2,237,914	18.7%	17.3%	16.6%
65-74	1,717,024	14.4%	15.1%	21.2%
75 plus	581,466	4.9%	5.5%	
Education				
Less than high school	69,674	2.6%	3.6%	12.0%
High school or equivalent	394,529	14.9%	17.1%	27.1%
Some college, no degree	638,111	24.1%	25.5%	20.4%
Associate’s degree	293,350	11.1%	10.7%	8.5%
Bachelor’s degree	670,457	25.4%	24.1%	19.7%
Graduate or professional degree	577,237	21.9%	19%	12.3%
Region				
Northeast	2,087,828	17.5%	17.6%	17.4%
Midwest	2,962,536	24.8%	21.1%	20.8%
South	4,368,172	36.6%	37.5%	38.0%
West	2,517,211	21.1%	23.8%	23.8%

Household size			
1	2,556,693	24%	21.7%
2	2,866,815	26.9%	27.3%
3-5	4,395,457	41.2%	41.9%
6-10	785,205	7.4%	8.4%
>10	62,604	0.5%	0.7%
