# Supplementary material for: Mental health during the COVID-19 pandemic: Impacts of disease, social isolation, and financial stressors
Source: PLoS One. 2022 Nov 23;17(11):e0277562. doi: 10.1371/journal.pone.0277562 (PMC9683625; doi:10.1371/journal.pone.0277562)
Supplement: S2 Table — (PDF) [file pone.0277562.s002.pdf]

Table S2. Mediation Analysis Predicting Mental Distress from County-level and Respondent-level Variables

		Mental distress														
		Total Effect					Direct Effect					Indirect Effect				
**Level**	**Predictor**	β	S.E.	t	p	95% C.I.	β	S.E.	t	p	95% C.I.	β	S.E.	t	p	95% C.I.
County	Disease threat	.013	.0005	29.82	0.000	.0126-.0143	.005	.0004	11.29	0.000	.0041-.0058	.009	.0001	87.94	0.000	.0083-.0087
County	Social distancing	.013	.0008	15.07	0.000	.0108-.0141	.006	.0008	6.9	0.000	.004-.007	.007	.0001	48.13	0.000	.0066-.0071
County	Financial threat	.004	.0004	9.6	0.000	.0033-.005	-.005	.0004	-10.73	0.000	-.005--.0038	.009	.0001	117.87	0.000	.0086-.0089
Respondent	Disease worry	.205	.0005	434.31	0.000	.2036-.2055	.205	.0005	434.31	0.000	.2036-.2055	NA	NA	NA	NA	NA
Respondent	Social contact	-.042	.0005	-91.69	0.000	-.043--.041	-.042	.0005	-91.69	0.000	-.043--.041	NA	NA	NA	NA	NA
Respondent	Financial worry	.158	.0005	349.68	0.000	.1572-.159	.158	.0005	349.68	0.000	.1572-.159	NA	NA	NA	NA	NA
Respondent	Age	-.149	.0003	-512.93	0.000	-.1491--.1479	-.138	.0003	-480.49	0.000	-.1387--.1375	-.01	.0001	-92.39	0.000	-.0106--.0102
Respondent	Female	.314	.0009	361.6	0.000	.3125-.3159	.208	.0009	240.65	0.000	.2062-.2096	.106	.0003	324.86	0.000	.1056-.1069
Respondent	Live with someone	-.048	.0009	-51.5	0.000	-.05--.0466	-.051	.0009	-57.37	0.000	-.0529--.0494	.003	.0003	9.24	0.000	.0022-.0034
Interaction	Social distancing X live with someone	-.002	.0009	-1.7	0.089	-.0034-.0002	-.002	.0009	-1.7	0.089	-.0034-.0002	NA	NA	NA	NA	NA

Interaction between *social distancing* and *live with someone* was included in the SEM model.
